# Professional Pebbles

**Published:** 1854-03

**Authors:** J. E. Sanborn


### IOWA MEDICAL JOURNAL.

VOL. I. KEOKUK, IOWA, MARCH, 1854. NO 8.

### ORIGINAL COMMUNICATIONS.

### PROFESSIONAL PEBBLES.

*Picked up on the Sea-Shore of Medical Science.*

BY J. E. SANBORN, M. D.

Those parts of the body which are newly formed, as for instance, a recent cicatrix, are much more liable to ulceration than those which have long existed.....Parts remote from the heart ulcerate more rapidly than those in its vicinity, from the enfeebled capillary forces. ....In searching for the causes of disease, we often err in limiting our observation to recent influences, quite omitting more remote but perhaps equally efficacious ones.....Slight causes, long continued, are often as efficient agents of disease, as sudden, but violent ones.....As a general thing, in case of severe internal inflammations, the sudden change of the pulse from very slow to very fast, is one of the first indications of the yielding of the system, and of approaching dissolution.....If the pupil be dilated during sleep, there is reason for fear. ....In obscure diseases of children, keep a sharp look out for some form of cerebral disorder, very likely hydrocephalus.....It is often very important to diagnose the *cause* of couvulsions, occurring to children, whether in the brain, or in some other organ. We may conclude it is *not* in the brain, if the attack be sudden, if it occur in the midst of previous health, if consciousness exist between the convulsions and if the respiration be not abnormal.

When consumption is inherited, it more generally developes itself in the same sex as that of the predisposing parent....In very young infants, under six months of age, the pulse is often quite undisturbed by dangerous illness, especially by serious abdominal affections.....In case of disease of any internal organ, produced by severe external injury, the longer the interval between the accident and the manifestation of disease, the less favorable will be the probabilities of recovery.....There are states of debility, in which the action of the heart, instead of being less frequent, is more frequent than in health, and the success of treatment will at once show itself accordingly.....When profuse perspiration occurs in the couse of protracted disease, without a crisis, the case demands much care ; exhaustion will very likely set in, and stimulants will be needed.....It is not the mere perspiration in fever, that is so salutary; none but out-siders think so, but it is that peculiarly favorable condition of the system which involves spontaneous perspiration The point is not to *make* the patient sweat, but to induce such a condition of the whole system that he will sweat himself.

The distressing and often fatal effects arising from severe constitutional irritation, generally depend much more upon the state of the system, than upon the amount or severity of local irritation, or even the absorption of a morbid poison.....Warm climates (and seasons) increase constitutional irritability and diminish the vital powers..... The treatment of chronic diseases frequently resolves itself into little else than the careful restoration of all the secretions, and the cautious removal of all obstruction in the pathway of the patient's spontaneous recovery.....An irritable constitution is more liable to the invasion of inflammation and in such, its course is usually more dangerous and extensive.....When leeches produce, as in some persons, a peculiar erythematous appearance, their use is not generally productive of favorable results.....It is in general and not partial paralysis that the preparations of nux vomica are usually most esteemed.....It is not new, but true, and apt to be overlooked, that functional disturbance is very likely to run into organic lesion.....Often in children, before death, there is a remisssion and sometimes a suspension of morbid manifestations—especially in cerebral diseases—a painful warning of impending dissolution.

In the earlier stages of acute bronchial inflammation, beware of opiates, not so much that they "check the expectoration," there is an error int hat notion, but because they aggravate the existing venous tendency, and may increase the disposition to coma.....We may say that in chronic bronchitis, the more profuse the expectoration, the less is the probability that the disease will finally become phthisis, especially if the sputa be albuminous—provided, of course no physical signs of consumption co-exist.....Hœmoptysis was one of the early symptoms of phthisis in two thirds of the cases which came under the observation of Louis: yet only three out of three hundred of his patients died of Hœmoptysis.

Hurried starting from sleep, with a sense of impending suffocation and a demand for fresh air, are frequently indicative of organic disease of the heart.....In cases of protracted dyspepsia, with general constitutional depression, but uncomplicated with positive gastric symptoms, it will be a wise precaution to look out for Phthisis.....It is alleged that convulsions in children are not caused by teething. *because* they do not cease upon incising the gums;—what of that? It is no new thing for sympathetic irritation to be kept up, long after the specific cause has been removed.....Any abnormal respiration, especially of a slow or irregular character, if unconnected with evidences of pulmonary disease, should at once direct attention to the brain.. ..In almost all diseases, the digestive function is more or less disordered; though it by no means follows that this derangement should be an object of treatment.....Mere symptoms are capricious enough—sometimes a swarm of them, with no corresponding disease: and again, the severest diseases, with hardly a manifestation. Their importance is by no means in proportion to their prominence. The most violent are often valueless, while the most obscure may render to us the greatest service.....It is a truth, often of practical value, that disease is seldom limited to one organ, or even to one portion of the system.....In the whole range of disease, there is hardly any one symptom, which, by itself, is always indicative of any one constant condition of an organ.....When in the course of disease, sympathetic irritations, as of the nervous, vascular or respiratory systems, are uncommonly well marked, the ordinary local symptoms of the disease are less manifest: but we must not infer from this that the primary disorder has yielded.....In treating the tympanitis of gastro-entritis, withold the internal administration of terebinthinates while the case admits of antiphlogistic treatment.....A celebrated English author states that he has never seen a case of acute inflammation of the liver, accompanied with pain in the shoulder; and Andral testifies that it is very seldom met with.....It is a pretty constant law, that in inflammation of the viscera, the greater or less pain indicates, with considerable accuracy, the superficial or deep seated locality of the inflammation.

